# Active modulation of visible light with graphene-loaded ultrathin metal plasmonic antennas

**DOI:** 10.1038/srep32144

**Published:** 2016-08-26

**Authors:** Renwen Yu, Valerio Pruneri, F. Javier García de Abajo

**Affiliations:** 1 ICFO-Institut de Ciencies Fotoniques, The Barcelona Institute of Science and Technology, 08860 Castelldefels (Barcelona), Spain; 2 ICREA-Institució Catalana de Recerca i Estudis Avançats, Passeig Lluis Companys 23, 08010 Barcelona, Spain

## Abstract

Electro-optical modulation of visible and near-infrared light is important for a wide variety of applications, ranging from communications to sensing and smart windows. However, currently available approaches result in rather bulky devices, suffer from low integrability, and can hardly operate at the low power consumption levels and fast switching rates required by microelectronic drivers. Here we show that planar nanostructures patterned in ultrathin metal-graphene hybrid films sustain highly tunable plasmons in the visible and near-infrared spectral regions. Strong variations in the reflection and absorption of incident light take place when the plasmons are tuned on- and off-resonance with respect to externally incident light. As a result, a remarkable modulation depth (i.e., the maximum relative variation with/without graphene doping) exceeding 90% in transmission and even more dramatic in reflection (>600%) is predicted for graphene-loaded silver films of 1–5 nm thickness and currently attainable lateral dimensions. These new structures hold great potential for fast low-power electro-optical modulation.

Collective conduction electron oscillations in metals (plasmons) provide the means to locally enhance the electromagnetic field of light within nanometer-scale regions, well below the optical wavelength^1^. In particular, plasmons in noble metal nanostructures can resonate at visible and near-infrared (vis-NIR) frequencies, thus holding great potential for photonic technologies. This has stimulated a great deal of work over the last decade aiming to tailor the spatial and spectral properties of plasmons^2^, with applications such as metamaterial design^3^,^4^, optical biosensing^5^,^6^,^7^, spectrophotometry^8^,^9^,^10^, photocatalysis^11^,^12^,^13^,^14^,^15^,^16^, and coloring^17^,^18^,^19^,^20^,^21^,^22^. Control over the plasmon characteristics generally relies on the design of optimized nanostructure composition and morphology. However, active control through external stimuli based on these principles is limited to relatively long response times, which are nonetheless useful for tunable metamaterial designs^23^, including externally driven phase transitions^24^,^25^. This scenario has substantially evolved with the advent of two-dimensional crystals, which can undergo radical changes in their optical response under electrical doping^26^,^27^,^28^. For example, electrical modulation of graphene plasmons has been demonstrated to produce octave-scale frequency shifts^29^. This potential has been recently exploited to improve optical sensing^7^. Unfortunately, intrinsically fast plasmon-based modulation has only been demonstrated at long wavelengths within or beyond the midinfrared (mid-IR) region, while its extension to shorter wavelengths imposes severe demands on the level of doping and the reduction in size of the structures, the combination of which still remains a challenge.

A possible strategy to achieve vis-NIR light modulation consists in exploiting the switching off of graphene absorption when it transits from undoped to doped states: indeed, undoped graphene absorbs 2.3% of the incident light over the vis-NIR range^30^,^31^; in contrast, when doped to a Fermi energy *E*_F_, an optical gap of size 2*E*_F_ opens up in which absorption is drastically reduced^26^,^27^. Electrical gating has been used to show *E*_F_ ~ 1 eV doping^27^, which in principle enables intrinsically fast vis-NIR light modulation. However, the poor amount of absorption produced by this material is limited by its atomic thickness, and consequently, order-one modulation requires coupling it to either strong optical resonances or large structures in which a substantial effect builds up. The latter strategy has been followed to demonstrate active modulation in the NIR light transmitted through graphene-loaded waveguides^32^ and photonic crystals^33^,^34^, although the resulting structures are rather bulky and extend over many optical wavelengths. Alternative approaches have been suggested, including coupling to Mie modes, lattice singularities in periodically patterned surfaces, and Fabry-Perot resonances^35^, which again require relatively bulky structures. More compact designs can be made by coupling to plasmonic particles, as recently demonstrated in the mid-IR, taking advantage of the relatively low absorption of noble metals in that spectral region, where graphene can make a big difference^36^,^37^,^38^,^39^,^40^. However, the small thickness of graphene is still a key limitation that prevents the extension of these methods toward the visible regime. This suggests the possibility of reducing the relative volume of the plasmon-supporting metal, which can in fact go down to a single monolayer while retaining a large optical strength^41^.

Here, we show through realistic simulations that ultrathin graphene-metal hybrid films (UGMs) can undergo order-one modulation in vis-NIR light transmission and reflection. We present attainable designs consisting of 50 nm wide ribbons formed by a thin noble metal film of thicknesses in the 1–6 nm range supported on monolayer graphene. These results open a viable route toward fast electro-optical modulation within that technologically important frequency range.

## Results

**Figure 1:**
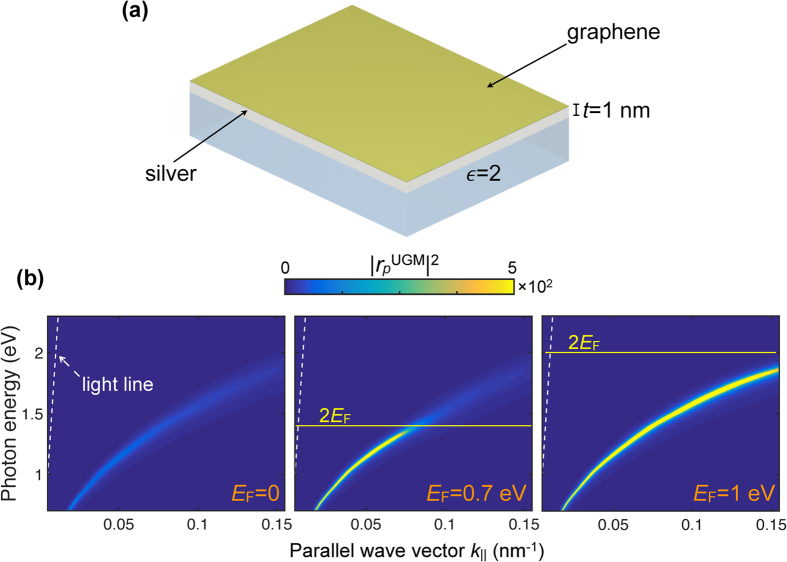
Tunable plasmon quenching in ultrathin graphene-metal hybrid films (UGMs). (**a**) Sketch of an UGM consisting of a graphene monolayer on top of an ultrathin silver film (thickness *t* = 1 nm), supported in turn by a silica substrate (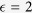). (**b**) Plasmon dispersion of the structure in (**a**) when the graphene is either undoped (*E*_F_ = 0, left) or highly doped (*E*_F_ = 0.7 eV, center; *E*_F_ = 1 eV, right), as illustrated by the photon-energy and parallel-wave-vector dependence of the reflectance for *p* polarization. Interband transitions produce strong plasmon quenching in the undoped structure and also in the doped structure when the photon energy exceeds 2*E*_F_ (i.e., above the yellow lines). We model graphene with the local-RPA conductivity^42^ assuming a mobility *μ* = 2000 cm^2^/(V s) throughout this work, while silver is described by its measured permittivity^46^. The light cone (dashed lines) is shown as a reference.

It is instructive to first discuss plasmons in homogeneous UGMs, whose dispersion relation can be conveniently obtained by plotting the reflectance for *p*-polarized light as a function of the parallel component of the wave vector *k*_||_ and photon energy. For the silica-supported sandwich structure depicted in Fig. 1a, the reflection coefficient 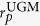 admits an analytical expression, with the optical properties of graphene and the metal modeled through their surface conductivity and permittivity, respectively (see Methods). We plot the resulting reflectance 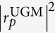 for doped and undoped graphene in Fig. 1b. Despite the relatively large thickness of the silver layer compared with the monolayer carbon film, it is clear that undoped graphene produces strong plasmon quenching via coupling to interband transitions^35^, while the optical gap opened in doped graphene leaves the plasmons nearly intact below ~2*E*_F_. Electron doping can thus modulate the plasmon strength dramatically in UGMs. Obviously, most of the region explored in Fig. 1b is far from the light cone, and it is indicative of what we should expect when plasmons are accessed by providing a source of additional momentum to externally incident light, for example by nanostructuring the film, as we discuss next.

**Figure 2:**
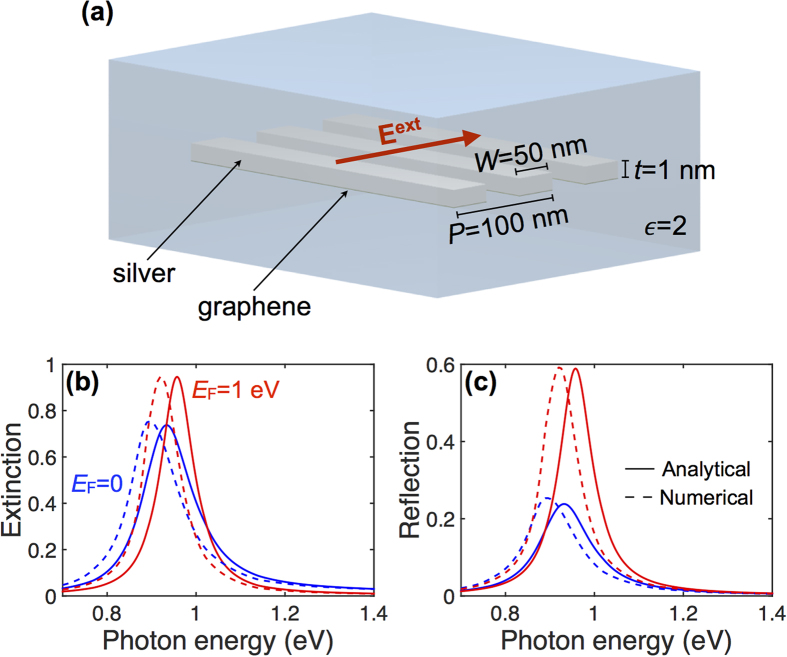
Optical switch based upon a periodic array of silver/graphene UGM ribbons. (**a**) Scheme of the structure under consideration. The ribbon array (metal thickness *t* = 1 nm, width *W* = 50 nm, period *P* = 100 nm, embedded in silica, 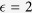) is illuminated under normal incidence with transversal polarization. (**b,c**) Extinction (**b**) and reflection (**c**) spectra for either doped (red curves, *E*_F_ = 1 eV) or undoped (blue curves, *E*_F_ = 0) graphene, showing ~26% (~36%) modulation depth in extinction (reflection) at 0.92 eV photon energy. Full numerical simulations (dashed curves) are in good agreement with analytical theory (solid curves, see Methods).

In order to more efficiently couple incident light to the UGM, we study the effect of patterning it into a periodic array of ribbons. These structures are simulated using a finite element method in the frequency domain (COMSOL), and the results compared to a simple analytical model presented in Methods. We consider the silica-embedded graphene-silver ribbon array shown in Fig. 2a (ribbon width *W* = 50 nm, array period *P* = 100 nm, silver thickness *t* = 1 nm) and study the extinction (1 − *T*) and reflection of normally-incident light polarized across the ribbons. The resulting spectra exhibit prominent features associated with the lowest-order dipolar mode of the ribbon^42^, roughly corresponding to the condition that the width *W* is half the plasmon wavelength in the planar film at that energy (i.e., *k*_||_ ~ *π*/*W* in Fig. 1b). The effect of doping is three-fold: (1) the plasmon peak is blue shifted, (2) the extinction and reflection maxima increase, and (3) the resonance line shape becomes narrower for larger *E*_F_. As a result, a modulation depth in extinction (reflection) ~26% (~36%) is observed at a photon energy ~0.92 eV when going from undoped graphene to *E*_F_ = 1 eV. The effects of doping are consistent with the suppression of damping channels (interband transitions) at photon energies below 2*E*_F_. Also, the noted blue shift is expected from the increase in available free carriers produced by doping. Remarkably, despite the simplicity of the analytical model (Fig. 2b, solid curves, see Methods), it is in excellent agreement with full numerical calculations (dashed curves), except for a small blue shift of the latter. In particular, the relative effect of doping is predicted to be the same for both types of simulations.

**Figure 3:**
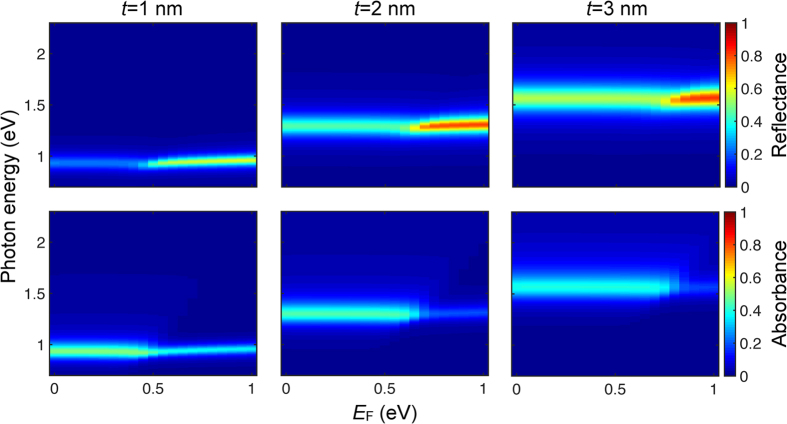
Tunable light modulation through UGM ribbon arrays. We show the normal-incidence reflectance (top) and absorbance (bottom) analytically calculated for the structure considered in Fig. 2a (graphene-silver ribbons in silica, width *W* = 50 nm, period *P* = 100 nm) with different metal thicknesses *t* = 1–3 nm (left to right) as a function of graphene Fermi energy (horizontal axis) and photon energy (vertical axis). Light polarization is across the ribbons.

Tunable light modulation in reflection and absorption for the class of structures sketched in Fig. 2a is a robust effect that takes place up to relatively large metal thickness, as we show in Fig. 3. These results clearly illustrate that an increase (decrease) in reflectance (absorbance) occurs when the Fermi energy exceeds approximately half the plasmon energy for each of the three metal thicknesses under consideration. Simultaneously, the plasmon resonance becomes narrower and slightly blue shifted in all cases. Importantly, together with the ribbon width, the silver thickness provides an extra degree of freedom to control the plasmon energy. Incidentally, the absorbance for *t* = 1 nm and undoped graphene reaches the maximum value of 50% that is possible for optically thin films^43^. Additionally, the modulation depth inferred from these results can be as high as ~23% in absorption and ~59% in reflection.

**Figure 4:**
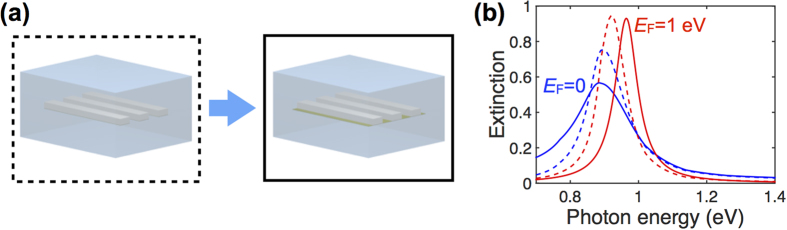
Optical switch based upon a periodic array of ultrathin silver ribbons on a continuous graphene sheet. We consider the effect of replacing the bottom graphene ribbons by a continuous monolayer graphene sheet (**a**) and compare the normal-incidence extinction for doped (*E*_F_ = 1 eV) and undoped (*E*_F_ = 0) graphene in both configurations (**b**). The continuous graphene film (solid curves) produces larger modulation depth than the graphene ribbons (dashed curves). Light polarization is across the ribbons. The ribbon thickness, width and period are *t*=1 nm, *W* = 50 nm and *P* = 100 nm, respectively.

A continuous graphene layer can be advantageous for actual implementations of these ideas, and therefore, we consider the structure shown in the solid box of Fig. 4a as a possible replacement for the one in the dashed box. For this new configuration our numerical simulations (Fig. 4b, solid curves) predict larger modulation depth (~55% at ~0.96 eV) than in the structured graphene (dashed curves). This can be understood as the result of additional optical attenuation in the inter-ribbon regions for undoped graphene. Simultaneously, the doped structure experiences a slightly larger blue shift, also produced by extra polarization due to the inter-ribbon regions.

**Figure 5:**
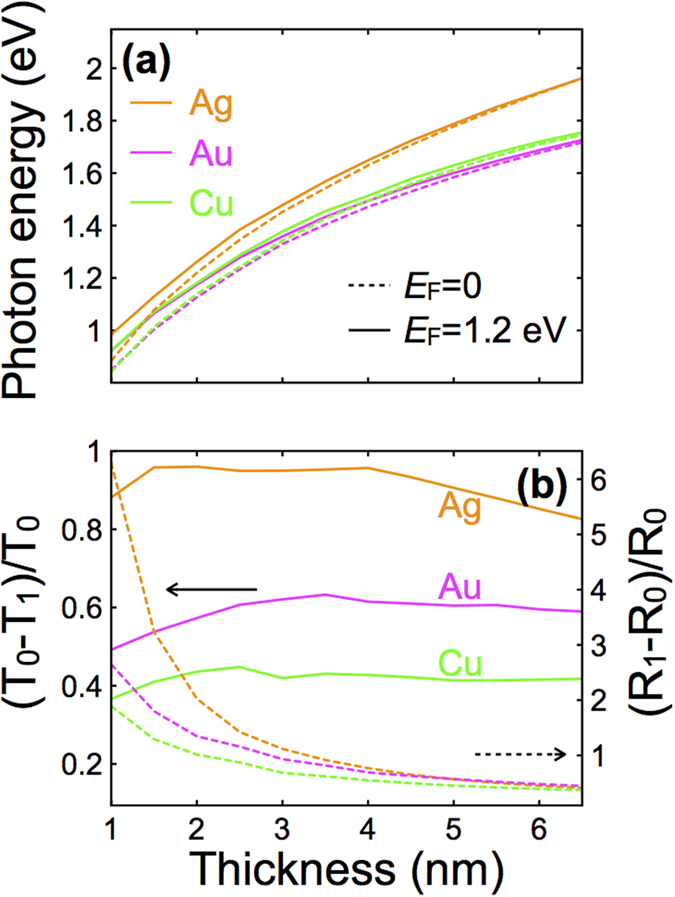
Dependence of light modulation on the choice of metal and metal thickness. We consider the same geometry as in the solid box of Fig. 4a (continuous graphene sheet, *W* = 50 nm, *P* = 100 nm). (**a**) Peak extinction energy with highly doped (solid curves) and undoped (dashed curves) graphene as a function of metal thickness for different noble metals. (**b**) Metal thickness dependence of the modulation depth for transmission (solid curves, left scale) and reflection (dashed curves, right scale), defined in terms of the normal-incidence, *p*-polarization transmittance and reflectance coefficients for undoped (*T*_0_ and *R*_0_) and doped (*T*_1_ and *R*_1_) graphene, evaluated at the peak photon energy of the doped structure.

Although silver is the less lossy of the noble metals in the spectral region under consideration, gold and copper can also do a fairly good job in UGMs. We present in Fig. 5a an overview of the plasmon energy corresponding to peak extinction as a function of metal thickness for these three different metals with and without doping. These results are obtained for the continuous-graphene structure of Fig. 4a (solid box) with *W* = 50 nm and *P* = 100 nm. The plasmon energy increases with thickness (i.e., with decreasing aspect ratio of the ribbons) and takes similar values for the three metals, as they also have similar conduction electron densities, although silver plasmons are slightly blue shifted because *d*-band screening is less efficient in this material. Incidentally, as the thickness increases, the structure is less sensitive to doping because graphene has to compete with a comparatively larger metal volume.

The modulation depth (Fig. 5b), defined as the relative change in transmission (or reflection) between doped and undoped structures (see vertical axis labels), is an important parameter for pondering potential applications. Obviously, this quantity degrades at large metal thickness, again because the weight of graphene is comparatively smaller, so it is overwhelmed by the metal. In the small thickness limit, the transmittance increases, also giving rise to a reduction in transmission modulation depth, while the modulation of reflection reaches values well above 1 for all three metals, and it approaches ~6 for silver. We thus conclude that optimum modulation in reflection is achieved for the thinnest metal films, while maintaining a reasonable modulation in transmission. Clearly, silver presents the best performance among the noble metals for vis-NIR light modulation, reaching a depth that exceeds 90% in transmission for metal thicknesses in the 1–5 nm range and >100% in reflection below 3 nm.

## Concluding Remarks

In summary, graphene can be used to modulate the optical response of thin metals, taking advantage of both the strong spectral weight of the resulting plasmons in the hybrid structure and the comparatively large volume of the carbon film when the metal thickness is reduced to only a few nanometers. Graphene doping causes a suppression of interband transitions, and in consequence, a reduction of plasmon damping, which is accompanied by blue shifts due to the addition of doping charges. We find a remarkable >90% modulation depth in transmission by using graphene-loaded silver ribbon arrays for metal thicknesses in the 1–5 nm range and lateral dimensions of tens of nanometers, which are attainable with currently available lithographies. The modulation of reflection is even more dramatic for metal thickness below 3 nm. The plasmon energies cover the 1–1.8 eV photon energy interval for these thicknesses, thus enabling the design of wide-spectral-range devices. Additionally, a continuous graphene layer appears to be advantageous to increase the modulation depth, and thus, only the thin metal film needs to be patterned.

Our proposed structures could be doped by contacting the ribbons at a large distance from the region in which they are intended to produce light modulation; the ribbons would then act as a top gate, with the graphene placed in the lower side, facing a bottom gate (e.g., a doped silicon substrate with an oxide layer); the charge induced in the top gate is then mainly contained within the outermost atomic layer of the conducting structure^44^. Working at a high intrinsic doping (e.g., as obtained by chemical doping) should allow us to modulate high photon energies by just swapping 2*E*_F_ across the desired spectral region. Additionally, the moderate amount of doping charges involved should enable the design of low power consumption devices. These findings open a new avenue for the development of compact electro-optical components such as tunable light filters, switchers, and sensors in the vis-NIR spectral region.

## Methods

### Graphene Conductivity

We model graphene using the local-RPA conductivity^42^,^45^


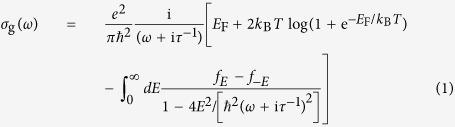


where 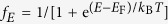 is the Fermi-Dirac distribution. Throughout this work, we assume room temperature (*T* = 300 K) and an inelastic decay time 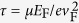 calculated for a mobility *μ* = 2000 cm^2^/(V s) and Fermi velocity *v*_F_ ≈ 10^6^ m/s.

### Reflection Coefficient of a Continuous UGM

The *k*_||_-dependent reflection coefficient 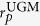 for *p*-polarized light incident on the sandwich structure of Fig. 1a can be simply constructed from the air-graphene-metal reflection (
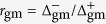
) and transmission (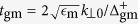) coefficients, the metal-graphene-air reflection (
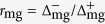
) and transmission (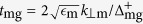) coefficients, and the metal-silica reflection coefficient (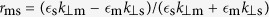). Here 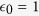, 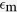, and 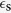 are the permittivities of air, metal, and silica, respectively; 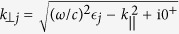 is the normal wave vector in medium *j* = 0, m, s, with the square root chosen such that Im{*k*_⊥*j*_} > 0; and we have defined 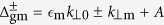, 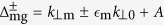, and *A* = 4*πσ*_g_*k*_⊥0_*k*_⊥m_/*ω*. From a simple multiple-scattering analysis, we find 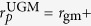 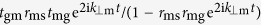, where *t* is the metal thickness.

### Analytical Model for the Optical Response of UGM Ribbons

We approximate the transversal ribbon polarizability per unit length *α* by the contribution of the lowest-order dipolar plasmon, which yields^42^


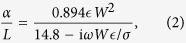


where *W* is the ribbon width, 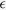 is the permittivity of the homogeneous environment, and the surface conductivity *σ* = *σ*_m_ + *σ*_g_ is the sum of metal and graphene contributions. The latter is given by (1), while we calculate the former as


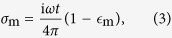


where *t* and 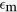 are the thickness and permittivity of the ultrathin metal. We take 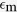 from optical measurements^46^.

### Analytical Model for Ribbon Arrays

Under the conditions of Fig. 2a, the reflection and transmission coefficients of the array for normal-incidence *p*-polarized light are^29^,^42^


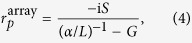



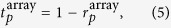


where 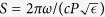, 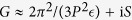, and *P* is the array period.

## Additional Information

**How to cite this article**: Yu, R. *et al*. Active modulation of visible light with graphene-loaded ultrathin metal plasmonic antennas. *Sci. Rep.* **6**, 32144; doi: 10.1038/srep32144 (2016).

## References

[b1] Li K. R., Stockman M. I. & Bergman D. J.. Self-similar chain of metal nanospheres as an efficient nanolens. Phys. Rev. Lett. 91, 227402 (2003). 14683271 10.1103/PhysRevLett.91.227402

[b2] Liz-Marzán L. M.. Tailoring surface plasmon through the morphology and assembly of metal nanoparticles. Langmuir. 22, 32–41 (2006). 16378396 10.1021/la0513353

[b3] Shalaev V. M.. Optical negative-index metamaterials. Nat. Photon. 1, 41–48 (2007).

[b4] Cortes C. L., Newman W., Molesky S. & Jacob Z.. Quantum nanophotonics using hyperbolic metamaterials. J. Opt. 14, 063001 (2012).

[b5] Anker J. N. . Biosensing with plasmonic nanosensors. Nat. Mater. 7(6), 442–453 (2008). 18497851 10.1038/nmat2162

[b6] Lu G. . Live-cell sers endoscopy using plasmonic nanowire waveguides. Adv. Mater. 26, 5124–5128 (2014). 24866811 10.1002/adma.201401237

[b7] Rodrigo D. . Mid-infrared plasmonic biosensing with graphene. Science. 349, 165–168 (2015). 26160941 10.1126/science.aab2051

[b8] Knight M. W., Sobhani H., Nordlander P. & Halas N. J.. Photodetection with active optical antennas. Science. 332, 702–704 (2011). 21551059 10.1126/science.1203056

[b9] Liu Y. . Plasmon resonance enhanced multicolour photodetection by graphene. Nat. Commun. 2, 579 (2011). 22146398 10.1038/ncomms1589 PMC4235953

[b10] Chalabi H., Schoen D. & Brongersma M. L.. Hot-electron photodetection with a plasmonic nanostripe antenna. Nano Lett., 14, 1374–1380 (2014). 24502677 10.1021/nl4044373

[b11] Seh Z. W. . Janus au-tio2 photocatalysts with strong localization of plasmonic near-fields for efficient visible-light hydrogen generation. Adv. Mater. 24(17), 2310–2314 (2012). 22467121 10.1002/adma.201104241

[b12] Gu J., Zhang Y.-W. & Tao. F. Shape control of bimetallic nanocatalysts through well-designed colloidal chemistry approaches. Chem. Soc. Rev. 41, 8050 (2012). 23080555 10.1039/c2cs35184f

[b13] Mukherjee S. . Hot electrons do the impossible: Plasmon-induced dissociation of h_2_ on au. Nano Lett. 13, 240–247 (2013). 23194158 10.1021/nl303940z

[b14] Mubeen S. . An autonomous photosynthetic device in which all charge carriers derive from surface plasmons. Nat. Nanotech., 8, 247–251 (2013). 10.1038/nnano.2013.18 23435280

[b15] Clavero C.. Plasmon-induced hot-electron generation at nanoparticle/metal-oxide interfaces for photovoltaic and photocatalytic devices. Nat. Photon. 8, 95–103 (2014).

[b16] Mukherjee S. . Hot-electron-induced dissociation of h2 on gold nanoparticles supported on sio2. J. Am. Chem. Soc. 136, 64–67 (2014). 24354540 10.1021/ja411017b

[b17] Lee H.-S., Yoon Y.-T., Lee S.-s., Kim S.-H. & Lee K.-D.. Color filter based on a subwavelength patterned metal grating. Opt. Express. 15(23), 15457–15463 (2007). 19550831 10.1364/oe.15.015457

[b18] Xu T., Wu Y.-K., Luo X. & Guo L. J.. Plasmonic nanoresonators for high-resolution colour filtering and spectral imaging. Nat. Commun. 1, 59 (2010). 20975716 10.1038/ncomms1058

[b19] Inoue D. . Polarization independent visible color filter comprising an aluminum film with surface-plasmon enhanced transmission through a subwavelength array of holes. Appl. Phys. Lett. 98, 093113 (2011).

[b20] Si G. . Reflective plasmonic color filters based on lithographically patterned silver nanorod arrays. Nanoscale, 5, 6243–6248 (2013). 23685642 10.1039/c3nr01419c

[b21] Roberts A. S., Pors A., Albrektsen O. & Bozhevolnyi S. I.. Subwavelength plasmonic color printing protected for ambient use. Nano Lett. 14, 783–787 (2014). 24392819 10.1021/nl404129n

[b22] Yu R. . Structural coloring of glass using dewetted nanoparticles and ultrathin films of metals. ACS Photon. 3, 1194–1201 (2016).

[b23] Karvounis A., Ou J.-Y., Wu W., MacDonald K. F. & Zheludev N. I.. Nano-optomechanical nonlinear dielectric metamaterials. Appl. Phys. Lett. 107, 191110 (2015).

[b24] Gholipour B., Zhang J., MacDonald K. F., Hewak D. W. & Zheludev N. I.. An all-optical, non-volatile, bidirectional, phase-change meta-switch. Adv. Mater. 25, 3050–3054 (2013). 23625824 10.1002/adma.201300588

[b25] Waters R. F., Hobson P. A., MacDonald K. F. & Zheludev N. I.. Optically switchable photonic metasurfaces. Appl. Phys. Lett. 107, 081102 (2015).

[b26] Li Z. Q. . Dirac charge dynamics in graphene by infrared spectroscopy. Nat. Phys. 4, 532–535 (2008).

[b27] Chen C. F. . Controlling inelastic light scattering quantum pathways in graphene. Nature. 471, 617–620 (2011). 21412234 10.1038/nature09866

[b28] Santos E. J. G. & Kaxiras E.. Electrically driven tuning of the dielectric constant in mos_2_ layers. ACS Nano, 7, 10741–10746 (2013). 24215099 10.1021/nn403738b

[b29] Fang Z. . Gated tunability and hybridization of localized plasmons in nanostructured graphene. ACS Nano, 7, 2388–2395 (2013). 23390960 10.1021/nn3055835

[b30] Nair R. R. . Fine structure constant defines visual transparency of graphene. Science. 320, 1308 (2008). 18388259 10.1126/science.1156965

[b31] Mak K. F. . Measurement of the optical conductivity of graphene. Phys. Rev. Lett. 101, 196405 (2008). 19113291 10.1103/PhysRevLett.101.196405

[b32] Liu M. . A graphene-based broadband optical modulator. Nature. 474, 64–67 (2011). 21552277 10.1038/nature10067

[b33] Gan X. . Strong enhancement of light–matter interaction in graphene coupled to a photonic crystal nanocavity. Nano Lett. 12, 5626–5631 (2012). 23043452 10.1021/nl302746n

[b34] Majumdar A., Kim J., Vuckovic J. & Wang F.. Electrical control of silicon photonic crystal cavity by graphene. Nano Lett. 13, 515–518 (2013). 23286896 10.1021/nl3039212

[b35] Yu R., Pruneri V. & García de Abajo F. J.. Resonant visible light modulation with graphene. ACS Photon. 2, 550–558 (2015).

[b36] Emani N. K. . Electrically tunable damping of plasmonic resonances with graphene. Nano Lett. 12, 5202–5206 (2012). 22950873 10.1021/nl302322t

[b37] Yao Y. . Broad electrical tuning of graphene-loaded plasmonic antennas. Nano Lett. 13, 1257–1264 (2013). 23441688 10.1021/nl3047943

[b38] Li Z. & Yua N.. Modulation of mid-infrared light using graphene-metal plasmonic antennas. Appl. Phys. Lett. 102, 131108 (2013).

[b39] Mousavi S. H. . Inductive tuning of fano-resonant metasurfaces using plasmonic response of graphene in the mid-infrared. Nano Lett. 13, 1111–1117 (2013). 23398172 10.1021/nl304476b

[b40] Emani N. K. . Electrical modulation of fano resonance in plasmonic nanostructures using graphene. Nano Lett. 14, 78–82 (2014). 24303876 10.1021/nl403253c

[b41] Manjavacas A. & García de Abajo F. J.. Graphene plasmonics: Challenges and opportunities. Nat. Commun. 5, 3548 (2014). 24671020 10.1038/ncomms4548

[b42] García de Abajo F. J.. Graphene plasmonics: Challenges and opportunities. ACS Photon. 1, 135–152 (2014).

[b43] Thongrattanasiri S., Koppens F. H. L. & García de Abajo F. J.. Complete optical absorption in periodically patterned graphene. Phys. Rev. Lett. 108, 047401 (2012). 22400887 10.1103/PhysRevLett.108.047401

[b44] Lang N. D. & Kohn W.. Theory of metal surfaces: Induced surface charge and image potential. Phys. Rev. B. 7, 3541–3550 (1973).

[b45] Gusynin V. P., Sharapov S. G. & Carbotte J. P.. On the universal ac optical background in graphene. New J. Phys. 11, 095013 (2009).

[b46] Johnson P. B. & Christy R. W.. Optical constants of the noble metals. Phys. Rev. B. 6, 4370–4379 (1972).

